# Oxidation Effects in Rare Earth Doped Topological Insulator Thin Films

**DOI:** 10.1038/srep22935

**Published:** 2016-03-09

**Authors:** A. I. Figueroa, G. van der Laan, S. E. Harrison, G. Cibin, T. Hesjedal

**Affiliations:** 1 Magnetic Spectroscopy Group, Diamond Light Source, Didcot, OX11 0DE, United Kingdom; 2 Clarendon Laboratory, Department of Physics, University of Oxford, Parks Road, Oxford, OX1 3PU, United Kingdom; 3 Department of Electrical Engineering, Stanford University, Stanford, CA 94305, USA; 4 Diamond Light Source, Didcot, OX11 0DE, United Kingdom

## Abstract

The breaking of time-reversal symmetry (TRS) in topological insulators is a prerequisite for unlocking their exotic properties and for observing the quantum anomalous Hall effect (QAHE). The incorporation of dopants which exhibit magnetic long-range order is the most promising approach for TRS-breaking. REBiTe_3_, wherein 50% of the Bi is substitutionally replaced by a RE atom (RE = Gd, Dy, and Ho), is a predicted QAHE system. Despite the low solubility of REs in bulk crystals of a few %, highly doped thin films have been demonstrated, which are free of secondary phases and of high crystalline quality. Here we study the effects of exposure to atmosphere of rare earth-doped Bi_2_(Se, Te)_3_ thin films using x-ray absorption spectroscopy. We demonstrate that these RE dopants are all trivalent and effectively substitute for Bi^3+^ in the Bi_2_(Se, Te)_3_ matrix. We find an unexpected high degree of sample oxidation for the most highly doped samples, which is not restricted to the surface of the films. In the low-doping limit, the RE-doped films mostly show surface oxidation, which can be prevented by surface passivation, encapsulation, or *in-situ* cleaving to recover the topological surface state.

Magnetically doped topological insulators (TIs) with broken time-reversal symmetry have been the focus of many recent studies^1^,^2^,^3^,^4^,^5^, as they exhibit exotic quantum and magneto-electric effects^6^,^7^, such as the quantum anomalous Hall effect (QAHE)^8^, and offer the prospect of potential applications in spintronic devices^9^. On the one hand, a relatively straightforward strategy to introduce magnetism in TIs is to dope them with transition metal (TM) elements. For example, the incorporation of Cr, Co, Mn, Fe, Ni, and Cu in the 3D-TI Bi_2_Se_3_ has been studied by extended x-ray absorption fine structure (EXAFS)^10^,^11^. On the other hand, one can follow a different approach by incorporating rare earth (RE) elements into the Bi chalcogenide lattice. RE ions offer the advantages of large magnetic moments (2–3 times larger than those of 3*d* TM doped systems) and, in thin films, a higher solubility^12^,^13^. Despite the interest in magnetically doped TIs for unlocking exotic physics, there is still controversy about the precise location of the magnetic dopants in the Bi chalcogenide lattice. Especially in the case of the TMs, e.g., Cr-doped Bi_2_Se_3_ (refs 14 and 15), the valence state of the dopant was not reliably known, until recently, when we were able to resolve it using x-ray absorption spectroscopies^16^.

A recent breakthrough in QAHE research has been the realization of perfectly quantized transport in the absence of an applied magnetic field at temperatures that can be reached without the need for a dilution refrigerator^17^. V-doping of (Bi,Sb)_2_Te_3_ has been shown to result in a hard ferromagnetic TI with a coercive field of ~1 T. Another promising system, as proposed by ab-initio calculations, are REBiTe_3_ compounds, i.e., a Bi_2_Te_3_ host in which 50% of the Bi atoms have been replaced by a RE such as Gd^18^. Gd-doping of bulk Bi_2_Te_3_ has been limited to ~5% in the past^19^,^20^, however, in a recent report up to 20% (in % of the Bi sites) has been demonstrated, resulting in an antiferromagnetic system^21^. In thin films, however, high concentrations of RE dopants can be incorporated into the Bi_2_Te_3_ matrix as thermal non-equilibrium deposition methods, such as molecular beam epitaxy (MBE), can be employed^12^. In case of Gd, a doping concentration of up to 40% (of the Bi sites) has been demonstrated, while maintaining high crystalline quality^12^.

Apart from the magnetic doping challenges, unintentional doping due to crystalline defects, such as Se vacancies in case of Bi_2_Se_3_ (ref. 22), is a crucial factor that determines the usefulness of TI films for device studies as well. Owing to increased bulk carrier densities, the topological surface state (TSS) can be fully suppressed in transport measurements^23^. For the exposure of Bi_2_Se_3_ films to ambient conditions it has been shown that a continuous growth of a native oxide leads to additional bulk carriers, overshadowing the relative contribution of the TSS^24^ and leading to a modification of the surface band structures^22^. For Bi_2_Se_3_ and Bi_2_Te_3_ films, Se^25^ and Te^26^ caps have shown to protect the as-grown surface well, and the TSS is recovered after ambient exposure followed by removal of the cap in ultra-high vacuum by heating. Another approach, most appropriate for surface-sensitive spectroscopic studies, is the *in-situ* cleaving of the TI films^27^.

The purpose of this study is the systematic investigation of the local electronic and structural environment of thin Bi_2_(Se, Te)_3_ films doped with the REs Gd, Dy, and Ho, after a prolonged air exposure. We use the element-specific technique of x-ray absorption fine structure (XAFS) to discern the location of the dopant in the Bi_2_(Se, Te)_3_ matrix, to determine the valence of the magnetic atoms, and to evaluate the oxidation levels, especially in the high-doping limit. We demonstrate that Gd, Dy, and Ho in the films are trivalent, which is consistent with isoelectronic substitution on Bi sites. EXAFS results at the RE *L*_3_ edges suggest effective incorporation of the RE atoms on Bi sites for low doping concentrations. For high RE concentrations, higher levels of oxygen are found (RE oxides). The trivalent character is in stark contrast to TM-doped films where, e.g., Cr, Mn, and Fe dopants were found to occupy octahedral sites and have divalent character when substituting trivalent Bi atoms in the Bi_2_Se_3_ matrix^11^. In the case of TM-doped samples, a local structural relaxation of the Bi_2_Se_3_ lattice is observed resulting in the contraction of the TM-Se distances, strengthening the covalent character of the bond^16^.

## Comparison of the Structural and Magnetic Properties

### Structural properties

**Figure 1:**
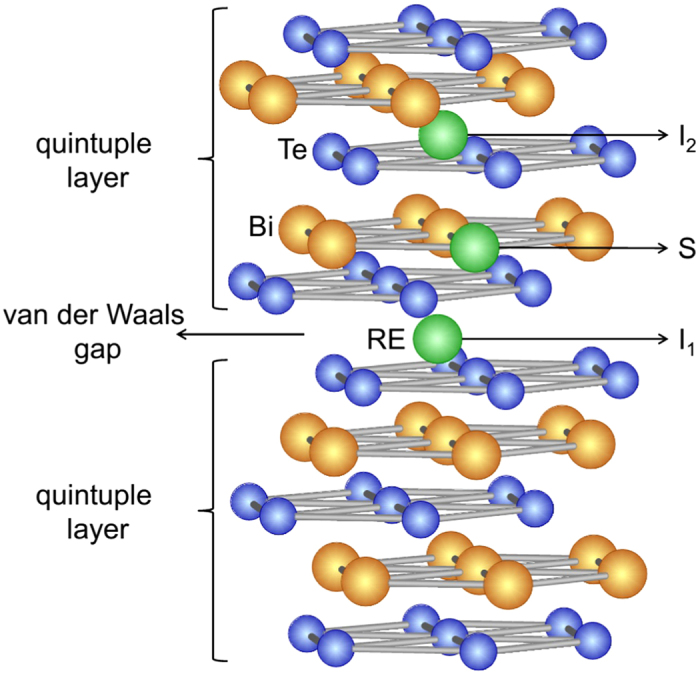
Crystal structure of Bi_2_Te_3_ (Bi = orange, Te = blue). Quintuple layers (Te-Bi-Te-Bi-Te) are weakly bonding across the van der Waals gap. RE dopants (green) are shown substituting for Bi (S), interstitially in the van der Waals gap (I_1_), and within the quintuple layer (I_2_).

**Figure 2:**
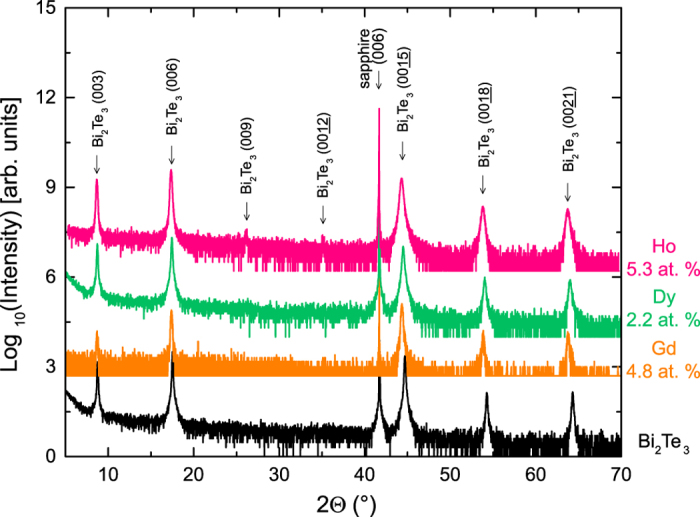
X-ray diffraction spectra for selected low-doping concentration Gd-, Dy-, and Ho-doped Bi_2_(Se, Te)_3_ samples. An undoped Bi_2_Te_3_ film is shown as a reference. Datasets have been vertically shifted for clarity.

The 3D-TI family of Bi_2_(Se, Te)_3_ compounds has a rhombohedral crystal structure with space group 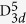 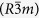. Figure 1 illustrates the layered structure of the crystal, with five atomic layers (Te–Bi–Te–Bi–Te) as a basic unit, known as a quintuple layer (QL). Bi_2_(Se, Te)_3_ thin films, doped with the RE elements Gd, Dy, and Ho, as described in detail in refs 13, 28 and 29, were grown by MBE on *c*-plane sapphire. Below a critical atomic percentage (at.% RE doping), the films were stoichiometric compounds with a 2:3 ratio of Bi and RE to Te (and Se, see Methods section for details). Judging from the shift and broadening of the film peaks observed in the x-ray diffraction (XRD) spectra, depicted in Fig. 2, a maximum RE concentration was determined, below which the films were rhombohedral, *c*-axis oriented, and free of peaks from secondary phases. In this case, the RE ion is isoelectronically (3+) substituting for Bi^3+^. The critical concentrations are ~16.4 at.% for Gd, ~14.2 at.% for Dy, and ~8.4 at.% for Ho. A summary of the investigated samples is shown in Table 1. Due to the low crystal quality, the *c*-axis lattice constant for the Ho sample above the critical concentration was not determined. The *c*-axis lattice constant increases upon doping for all dopants, and their values are very similar for Gd and Ho, and smaller for Dy. No direct dependency on the ionic radii is found [the ionic radii for 3+ ions (octahedral coordination) are 1.17 Å (Bi), 1.053 Å (Gd), 1.027 Å (Dy), and 1.015 Å (Ho)].

### Magnetic properties

The effective magnetic saturation moments for the 4*f* electrons of the Gd, Dy, and Ho doped TIs, as determined by x-ray magnetic circular dichroism (XMCD) at the *M*_4,5_ edges in an applied out-of-plane field, are listed in Table 2 (refs 13, 28 and 29), together with the theoretical values for the *LS*-coupled atomic ground states. The temperature-dependent XMCD plots reveal paramagnetic behaviour down to the lowest probed temperature of ~2 K. Table 2 shows that saturated moments obtained by XMCD at high field and near liquid He temperatures are 40–50% of the theoretical maximum values. While the explanation for this is beyond the scope of this paper, possible causes can be an antiferromagnetic alignment of the oxidized RE-ions, 4*f* crystal-field interaction, and non-collinear moments in the paramagnetic phase.

## Results and Discussion

**Figure 3:**
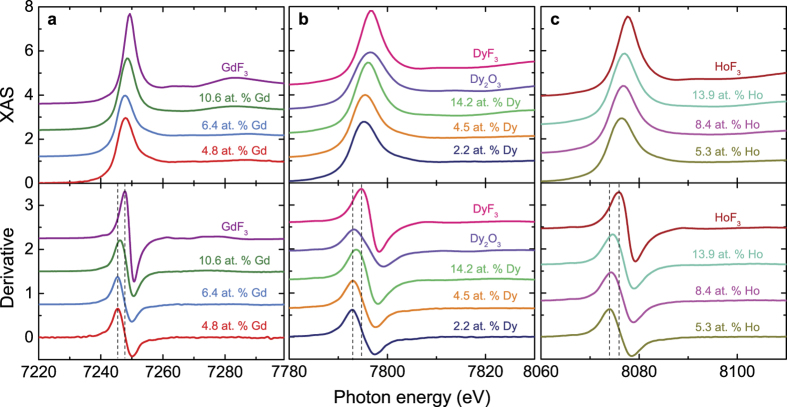
XANES spectra (top panels) and their first derivatives (bottom panels) at the RE *L*_3_ edge of (**a**) Gd; (**b**) Dy; and (**c**) Ho in RE-doped Bi_2_(Se, Te)_3_ thin films. For comparison the following reference standards are shown: (**a**) GdF_3_; (**b**) Dy_2_O_3_ and DyF_3_; and (**c**) HoF_3_. Both spectra and derivatives have been vertically shifted for clarity. Dotted lines mark the position of the maxima in the derivatives for the lowest RE concentration and the associated (RE)F_3_ standard.

X-ray absorption near edge structure (XANES) spectra of the RE-doped thin films are shown in Fig. 3. All samples show RE *L*_3_ spectra characteristic of a 3+ oxidation state. The absorption jump lies around those for the standards measured as reference, which are 3+ (i.e., GdF_3_, DyF_3_, Dy_2_O_3_, and HoF_3_). For all RE-doped films the white line intensity increases with increasing RE content in the sample.

Changes in the electronic state are better evaluated from the first derivative of the XANES spectrum, shown in the bottom panel of Fig. 3 for all RE-doped samples. The maximum of the first derivative is very close in energy for those Gd samples with concentration <10 at.%, which indicates a very similar electronic environment for the Gd atoms in these doped films. A shift toward higher energy (~0.8 eV) is observed for the sample with the highest Gd content (10.6 at.% Gd). In the case of Dy, samples with Dy content between 2.2–4.5 at.% have the maximum of their first derivative very close in energy; the sample with the highest amount of Dy (14.2 at.%) deviates from this trend since the position of the first derivative is about 0.8 eV higher than the rest of the samples. These XANES results indicate that there is a strong change in the electronic structure of the Gd and Dy atoms in the samples when going from 6.4 to 10.6 at.% Gd, and from 4.5 to 14.2 at.% Dy doping, respectively. In the case of Ho, there is a gradual energy shift in the maximum of their first derivative towards high energies with Ho content in the sample. A higher photon energy of the white line corresponds to a more electropositive rare earth ion^30^. Thus the RE fluoride is found at a higher energy than the oxide. The energy shift of the white line is also related to the nearest neighbour bond distance. These XANES results suggest that the average bond distance between the RE atoms and their nearest neighbours in the doped TIs, particularly for the case of Dy and Gd, is shorter for samples in the low doping regime (below 10%) than those of high doping concentration.

**Figure 4:**
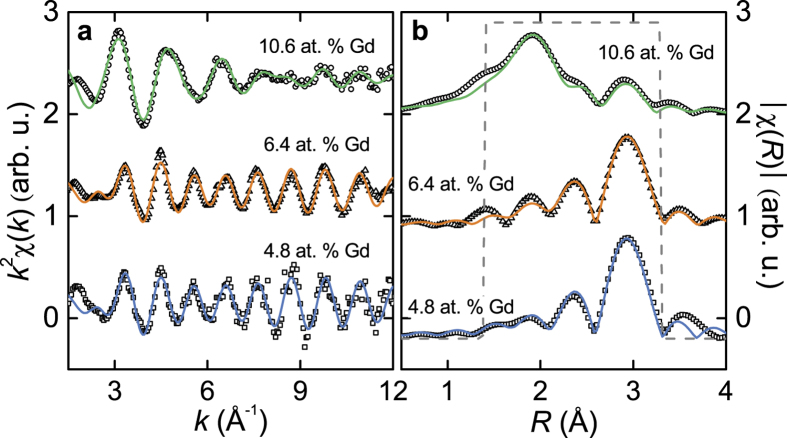
Gd-doped films. (**a**) EXAFS signal (open symbols) at the Gd *L*_3_-edge together with their best fits (solid lines). (**b**) Fourier transform of the EXAFS signal and fits in (**a**). The curves have been vertically shifted for clarity.

**Figure 5:**
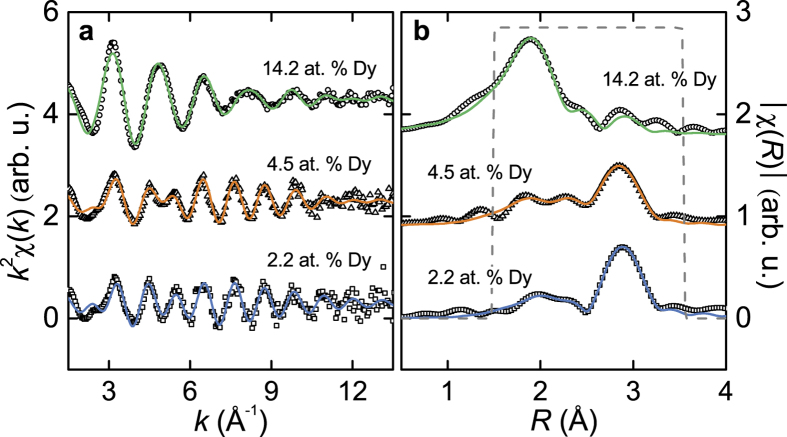
Dy-doped films. (**a**) EXAFS signal (open symbols) at the Dy *L*_3_-edge together with their best fits (solid lines). (**b**) Fourier transform of the EXAFS signal and fits in (**a**). The curves have been vertically shifted for clarity.

**Figure 6:**
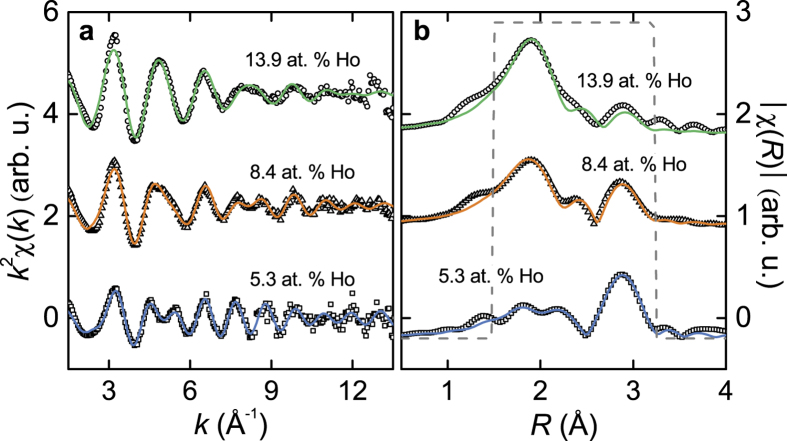
Ho-doped films. (**a**) EXAFS signal (open symbols) at the Ho *L*_3_-edge together with their best fits (solid lines). (**b**) Fourier transform of the EXAFS signal and fits in (**a**). The curves have been vertically shifted for clarity.

Fits of the RE *L*_3_-edge EXAFS signal were performed to extract information about bond distances and coordination of the RE dopants in the Bi_2_Te_3_ thin films. Figures 4, 5, 6 show the raw *χ*(*k*) EXAFS signal and module of the Fourier transform (FT) for Gd-, Dy-, and Ho-doped samples performed over a [3 − 11] Å^−1^, [3 − 12] Å^−1^, and [3 − 12] Å^−1^ *k*-range using a Hanning window function, and Δ*k* = 1, 2, and 1.5 Å^−1^, respectively. All plots are performed using a *k*^2^ weight.

Different models were tested to fit the EXAFS signal at the RE *L*_3_-edge. Fits were performed on *R*-space in a [1.4 − 3.3] Å, [1.5 − 3.5] Å, and [1.5 − 3.2] Å range for the Gd-, Dy-, and Ho-doped samples, respectively, using a Hanning window function, so that it covered the first coordination shell of the RE atoms [see Figs 4b–6b]. The best fits were achieved using a model that included different neighbours, thus fitting the relative fraction content in each sample. For Dy- and Ho-doped samples, RE-Te, RE-Se and RE-O bonds were used in the model, given that some amount of Se atoms is present in the samples; for Gd-doped films only Gd-Te and Gd-O were required for the fits. The parameters fitted were the interatomic distance (*R*), the Debye-Waller factor (*σ*^2^) and the relative fraction (*x*) for each scattering path. The latter was fitted so that it followed the expression *x*_Te_ + *x*_Se_ + *x*_O_ = 1 for Dy- and Ho- doped samples, and *x*_Te_ + *x*_O_ = 1 for the Gd-doped ones. The shift in the threshold energy (Δ*E*_0_) was allowed to vary for each sample. The amplitude reduction factor 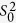 was set to that obtained for the fit of the respective RE standards (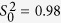, 0.98, and 0.94 for Gd, Dy, and Ho, respectively). Results for these fits are shown in Figs 4, 5, 6, and the parameters extracted for each sample are listed in Tables 3, 4, 5 for the Gd-, Dy-, and Ho-doped films, respectively. Other fit attempts revealed no contribution of RE–RE scattering paths in the first coordination shell.

For the Gd-doped samples with <10.6 at.% concentration, most of the Gd dopants (~72%) are found to be in an octahedral environment of Te atoms, which is consistent with substitutional incorporation into Bi sites. In fact, the Gd-Te bond near 3.08 Å agrees well with the Bi-Te of ~3.07 Å in the undoped Bi_2_Te_3_ crystal (ICSD 74348). Interstitial incorporation of the Gd atoms into the van der Waals gap would yield a similar octahedral environment but with a shorter Gd-Te bond distance near 2.88 Å. The rest of Gd dopants in these samples (~28%) are located in an oxygen octahedral environment, which suggests oxidation of some of the RE dopants. For the sample with 10.6 at.% Gd, most dopants are oxidized.

The local environment for the Dy atoms in the Dy-doped Bi_2_(Se, Te)_3_ films reveal coordination with Te, Se, and O neighbours. The Dy-Te and Dy-Se bonds are part of an octahedral environment and suggests effective incorporation of the Dy dopants into Bi sites of the lattice. A certain amount of Dy-oxide is observed for all samples, however, it is rather low for the least doped sample (around 9% Dy-oxide for 2.2 at.% Dy doping), but it increases for higher doping concentrations. For the most highly doped sample (14.2 at.% Dy) most of the dopants are oxidized.

Similarly to the Dy doping case, some of the Ho atoms in the Ho-doped Bi_2_(Se, Te)_3_ samples have Te, Se, and O neighbours. The Ho-Te (and Ho-Se) bond distances agree with substitutional incorporation of the Ho atoms into Bi sites. Also, the amount of oxygen in these Ho-doped samples increases with increasing Ho doping concentration.

Judging from the high crystalline quality of the films as determined by XRD right after growth, and the *N* = 6 coordination with oxygen, it can be concluded that the oxidation occurred post-growth, starting from the surface, and constitutes an ageing effect of the sample. The lower doping concentration samples have been successfully cleaved in ultra-high vacuum, following an in-air transfer, and the TSS has been successfully recovered^12^,^27^,^29^,^31^. The higher doping concentration samples, on the other hand, are oxidized to a large degree. Note that these samples are structurally of a slightly lower quality^13^,^28^,^29^,^31^. It appears that these crystalline imperfections, or the occurrence of accompanying strain, promote oxidation over time.

## Conclusions

In conclusion, we have performed a study of the local structure of Gd, Dy, and Ho atoms in the low and high doping limit in Bi_2_(Se, Te)_3_ thin films upon exposure to air. All RE dopants were found to be trivalent. For low doping concentrations, RE atoms effectively substitute Bi atoms in the lattice. Their surfaces are oxidized, however, the bulk of the films remains unaffected as seen, e.g., in ARPES measurements on *in-situ* cleaved samples. For higher doping concentrations above ~10 at.%, the samples are highly oxidized throughout their volume. Interestingly, the oxidation rate depends on the RE doping concentration, which is linked to the slightly lower structural quality. It has to be stressed, however, that the higher doped films do not show secondary phases or a stoichiometry that departs from the 2:3 ratio expected from substitutional incorporation of the RE on Bi sites. The films with lower RE concentrations are largely unaffected by the increased oxidation rate and can be simply cleaved *in-situ* after an in-air transport for surface-sensitive measurements^27^. In order to preserve the pristine surface, as needed for the nanofabrication of device structures, and to prevent or reduce oxidation, a Se or Te capping layer should be deposited *in-situ*^25^, which can be thermally removed right before the fabrication process.

## Methods

### Thin film sample preparation

The Bi_2_(Se, Te)_3_ thin films, doped with the RE metals Gd, Dy, and Ho, were grown by MBE on *c*-plane sapphire. During growth, a Te overpressure of ~15–20× was maintained with respect to the Bi flux, as measured by *in-situ* beam flux monitoring. Standard effusion cells were used for evaporation of Dy, Ho, and Bi. A high temperature effusion cell was used for Gd and a standard or cracker effusion cell was used for evaporation of Te. All elemental materials were of ultra-high purity (99.9999% for Bi and Te, 99.9% for Gd and 99.99% for Dy and Ho). The RE-doped Bi_2_Te_3_ films were grown using the established two-step growth method^32^. First, a nucleation layer was deposited at a temperature 50 °C lower than the final growth temperature, *T*_G_. This layer was then annealed under Te flux, while the temperature was ramped up to *T*_G_, where the growth then proceeded until the desired film thickness was achieved. The optimum growth temperature was slightly different for the three RE systems, with a *T*_G_ of 240 °C for Gd, and 300 °C for Dy and Ho. The typical effusion cell temperatures for the REs were 1250–1400 °C for Gd, 825–925 °C for Dy, and 840–900 °C for Ho. All doped films have thicknesses between 50–120 nm, except for the 1320 °C Gd film which is 500 nm thick. A detailed exposition of the growth process, structural and magnetic studies can be found elsewhere for Gd [refs 12 and 13], Dy [refs 28 and 31], and Ho [ref. 29], respectively. Note that the Gd-doped films were grown in a Se-free MBE system, whereas the Dy- and Ho-doped films show unintentional Se doping of up to 5 at.% due to elevated temperatures in the chamber during growth. For all samples, the combined Bi and RE to Te and Se ratio is, within error, 2:3, as expected from a substitutionally doped Bi_2_Te_3_ compound.

### Structural characterisation

The film quality was evaluated using *in-situ* reflection high-energy electron diffraction (RHEED) for the Dy and Ho films (cf. Supplementary Fig. 1), and *ex-situ* XRD for all films (cf. Supplementary Fig. 2). Sharp, streak-like RHEED patterns were observed for all high-quality samples, indicative of 2D growth^13^,^28^,^29^. Judging from the shift and broadening of the film peaks observed in the XRD spectra, depicted in Fig. 2, a maximum RE concentration was determined, below which the films were rhombohedral, *c*-axis oriented, and free of peaks from secondary phases. The film compositions were determined from a combination of Rutherford backscattering spectrometry (RBS) and particle-induced x-ray emission (PIXE).

### Magnetic characterisation

The electronic character of the magnetic ground state was determined using XMCD^33^. This element-specific technique is capable of unambiguously determining the electronic and magnetic state of transition metal and rare earth magnetic dopants in Tis^5^,^34^,^35^. X-ray absorption spectra (XAS) of the Gd, Dy, and Ho doped TIs were measured at the *M*_4,5_ edges on beamline I10 (BLADE) at the Diamond Light Source (Oxfordshire, UK) using a 14 T superconducting magnet. XAS measurements were made simultaneously in total-electron-yield and fluorescence yield modes. The XMCD is obtained from the difference between two XAS spectra recorded with the x-ray helicity vector and applied magnetic field anti-parallel and parallel, respectively^36^. The magnetic field is always parallel to the x-ray beam and along the sample (normal ‖ *c*-axis). The XMCD is measured by reversing the polarization of the incident x-rays to avoid changing the magnetic field of the superconducting magnet. The 4*f* magnetic moments were extracted from the XMCD spectra for Gd, Dy, and Ho as described in refs 13, 28 and 29, respectively.

### XAFS measurements

X-ray absorption near edge structure (XANES) and EXAFS spectra of the doped films were collected at room temperature at the Gd, Dy, and Ho *L*_3_ edges (~7243, 7790, and 8071 eV, respectively) on beamline B18 at the Diamond Light Source. A nine-element solid-state Ge detector with digital signal processing for fluorescence XAFS, high energy resolution, and high count rate was used to measure with the beam at 45° incidence with respect to the sample plane. All spectra were acquired in quick-EXAFS mode, using the Pt-coated branch of collimating and focusing mirrors, a Si(111) double-crystal monochromator and a pair of harmonic rejection mirrors. The photon energy range for each scan allowed us to extract information in the extended region up to *k* = (12 − 14) Å^−1^. On average, 35 scans were recorded per thin film. As references, GdF_3_, DyF_3_, Dy_2_O_3_, and HoF_3_ were also measured. The powdered reference samples were pressed into a pellet with the optimized quantity for measurements in transmission mode at their respective absorption edge.

### XAFS analysis

EXAFS spectra were processed and analysed using the various tools of the IFEFFIT XAFS package^37^. This involved preliminary reduction of the EXAFS raw data, background removal of the x-ray absorption data *μ*(*E*), conversion of *μ*(*E*) to *χ*(*k*), normalization and weighting scheme; all of them performed with AUTOBK and ATHENA. EXAFS data analysis and fitting on all references and samples were performed in ARTEMIS, making use of models based on crystallographic information obtained from the ICSD database. The atomic clusters used to generate the scattering paths for fitting were generated with atoms^38^.

## Additional Information

**How to cite this article**: Figueroa, A. I. *et al*. Oxidation Effects in Rare Earth Doped Topological Insulator Thin Films. *Sci. Rep.* **6**, 22935; doi: 10.1038/srep22935 (2016).

## Supplementary Material

Supplementary Information

## Figures and Tables

**Table 1:** Growth and structural parameters of the RE-doped Bi_2_(Se, Te)_3_ thin films.

RE	RE *T* (°C)	at.% RE	*c* (Å)
Gd	1280	4.8 ± 1.0	30.62 ± 0.01
	1320	6.4 ± 1.0	30.71 ± 0.10
	1360	10.6 ± 1.0	30.94 ± 0.01
Dy	850	2.2 ± 1.0	30.51 ± 0.01
	875	4.5 ± 1.0	30.52 ± 0.01
	925	14.2 ± 1.0	30.8 ± 0.1
Ho	850	5.3 ± 2.0	30.63 ± 0.02
	865	8.4 ± 2.0	30.73 ± 0.04
	875	13.9 ± 2.0	–

RE cell temperature, RE *T*, RE doping concentration as obtained from the combined Rutherford backscattering spectrometry (RBS) and particle-induced x-ray emission (PIXE) measurements, at.% RE, and *c*-axis parameter extracted from XRD measurements^13^,^28^,^29^. The *c*-axis lattice for undoped Bi_2_(Se, Te)_3_ was determined to be 30.42 Å.

**Table 2:** The spin, orbital, and total angular momenta, *S, L, J*, for the *LS*-coupled 4*f* ground state (GS) of the REs, with their corresponding effective magnetic 4*f* moment, 
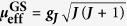
 (in *μ*_B_), using the Landé splitting factor, *g*_*J*_ = 3/2 + [*S*(*S* + 1) − *L*(*L* + 1)]/[2*J*(*J* + 1)].

RE	*S*	*L*	*J*	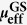	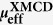	*x*_RE_	*μ*_0_*H*	*T*	ref.
Gd^3+^ 4*f*^7^	7/2	0	7/2	7.93	4.04	10.6	6	<5	13
Dy^3+^ 4*f*^9^	5/2	5	15/2	10.65	4.20	4.5	7	5	28
Ho^3+^ 4*f*^10^	2	6	8	10.6	4.50	5.5	7	2.5	29

Further shown are the experimental values, 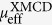 (in *μ*_B_/RE ion), obtained by XMCD for given concentration *x*_RE_ (in at.%), in an applied out-of-plane field *μ*_0_*H* (in T), at temperature *T* (in K) (see references).

**Table 3:** Structural parameters obtained from the Gd *L*_3_-edge EXAFS fits for the Gd-doped Bi_2_(Se, Te)_3_ thin films.

Bond	Parameter	at.% Gd		
		4.8	6.4	10.6
Gd-Te	*x*_Te_	0.72	0.72	0.14
*N* = 6	*R*_Gd-Te_ (Å)	3.08	3.09	3.09
	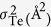	0.006	0.007	0.005
Gd-O	*x*_O_	0.28	0.28	0.86
*N* = 6	*R*_Gd-O_ (Å)	2.37	2.43	2.38
	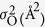	0.020	0.023	0.010
	Δ*E*_0_ (eV)	1.6	2.0	0.6

Coordination number, *N*, interatomic distance, *R*, Debye-Waller factor, *σ*^2^, and fraction *x*, for each path. The uncertainty in *x* is ±10%, in *R* ±0.02 Å, in Δ*E*_0_ ±0.2 eV, and in *σ*^2^ ±20%.

**Table 4:** Structural parameters obtained from the Dy *L*_3_-edge EXAFS fits for the Dy-doped Bi_2_(Se, Te)_3_ thin films.

Bond	Parameter	at.% Dy		
		2.2	4.5	14.2
Dy-Te	*x*_Te_	0.61	0.46	0.06
*N* = 6	*R*_Dy-Te_ (Å)	3.08	3.08	3.08
	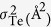	0.009	0.009	0.004
Dy-Se	*x*_Se_	0.30	0.32	–
*N* = 6	*R*_Dy-Se_ (Å)	2.83	2.84	–
	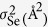	0.012	0.010	–
Dy-O	*x*_O_	0.09	0.22	0.94
*N* = 6	*R*_Dy-O_ (Å)	2.37	2.33	2.34
	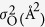	0.006	0.009	0.009
	Δ*E*_0_ (eV)	1.1	0.5	0.2

Coordination number, *N*, interatomic distance, *R*, Debye-Waller factor, *σ*^2^, and fraction *x*, for each path. The uncertainty in *x* is ±10%, in *R* ±0.02 Å, in Δ*E*_0_ ±0.2 eV, and in *σ*^2^ ±20%.

**Table 5:** Structural parameters obtained from the Ho *L*_3_-edge EXAFS fits for the Ho-doped Bi_2_(Se, Te)_3_ thin films.

Bond	Parameter	at.% Ho		
		5.3	8.4	13.9
Ho-Te	*x*_Te_	0.45	0.31	0.09
*N* = 6	*R*_Ho-Te_ (Å)	3.07	3.06	3.08
	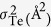	0.007	0.008	0.006
Ho-Se	*x*_Se_	0.11	–	–
*N* = 6	*R*_Ho-Se_ (Å)	2.79	–	–
	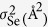	0.003	–	–
Ho-O	*x*_O_	0.44	0.69	0.91
*N* = 6	*R*_Ho-O_ (Å)	2.33	2.33	2.34
	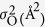	0.015	0.009	0.008
	Δ*E*_0_ (eV)	0.3	−0.2	0.9

Coordination number, *N*, interatomic distance, *R*, Debye-Waller factor, *σ*^2^, and fraction *x*, for each path. The uncertainty in *x* is ±10%, in *R* ±0.02 Å, in Δ*E*_0_ ±0.2 eV, and in *σ*^2^ ±20%.
