# Supplementary material for: Oxidation Effects in Rare Earth Doped Topological Insulator Thin Films
Source: Sci Rep. 2016 Mar 9;6:22935. doi: 10.1038/srep22935 (PMC4783712; doi:10.1038/srep22935)
Supplement: Supplementary Information [file srep22935-s1.doc]

Supporting Online Material for

**Oxidation Effects in Rare Earth Doped Topological Insulator Thin Films**

A. I. Figueroa,^1^ G. van der Laan,^1^ S. E. Harrison,^2, 3^ G. Cibin,^4^ and T. Hesjedal^2,4,*^

^1^Magnetic Spectroscopy Group, Diamond Light Source, Didcot, OX11 0DE, United Kingdom

^2^ Clarendon Laboratory, Department of Physics, University of Oxford, Parks Road,

Oxford, OX1 3PU, United Kingdom

^3^Department of Electrical Engineering, Stanford University, Stanford, California 94305, USA

^4^Diamond Light Source, Didcot, OX11 0DE, United Kingdom

**Reflection high-energy electron diffraction (RHEED) images and X-ray diffraction (XRD) spectra are provided for the investigated thin film samples in Supplementary Figures 1 and 2, respectively.**


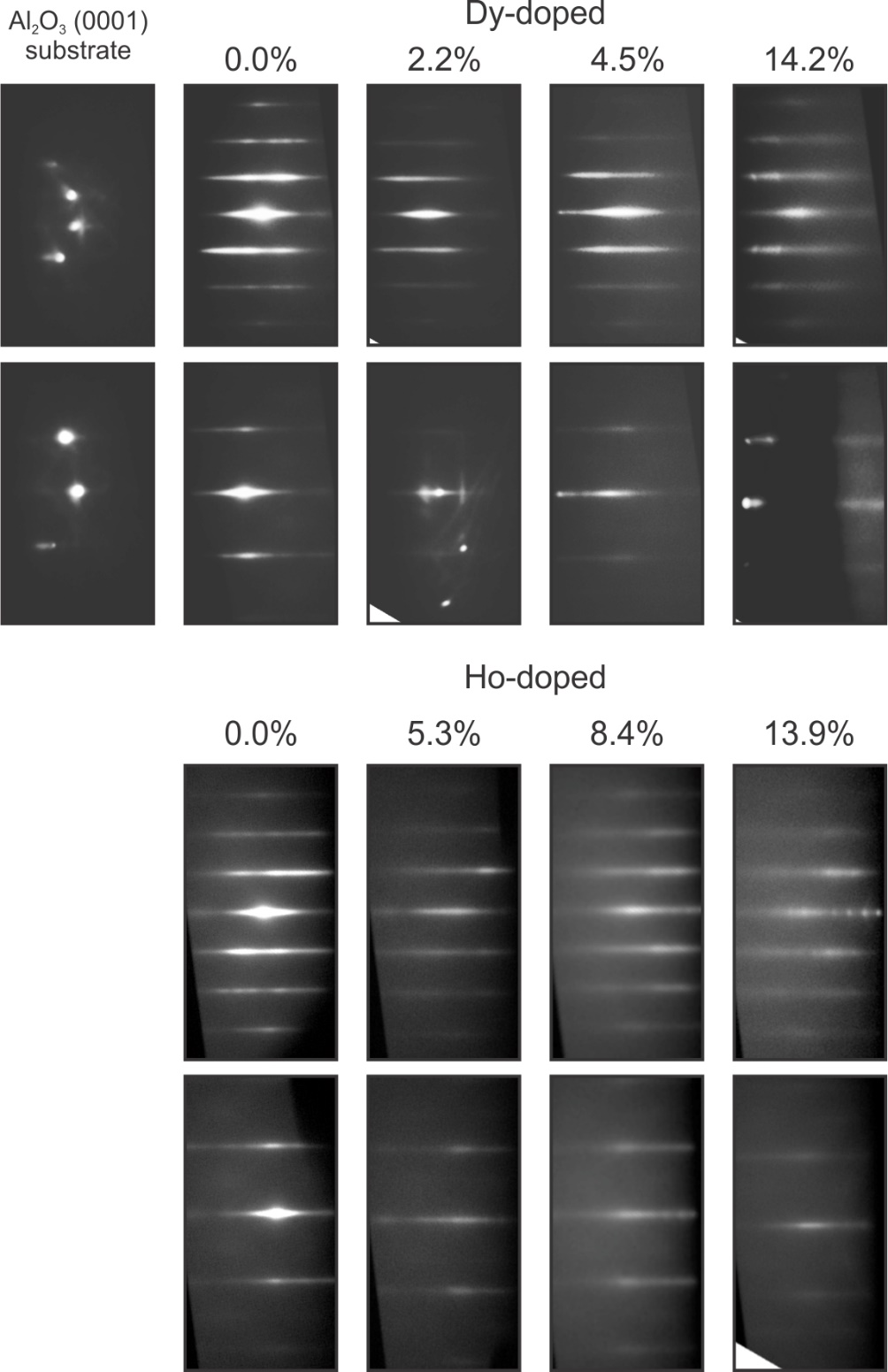


**Figure S1**. Reflection high-energy electron diffraction (RHEED) images of the Al_2_O_3_ (0001) substrate (top left), and the Dy-doped (upper rows) and Ho-doped (lower rows) Bi_2_(Se,Te)_3_ thin films. The patterns were recorded along the [10
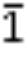
0] (above) and [11
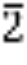
0] azimuth (below) of Al_2_O_3_ (0001) for the Dy and Ho concentrations as indicated.


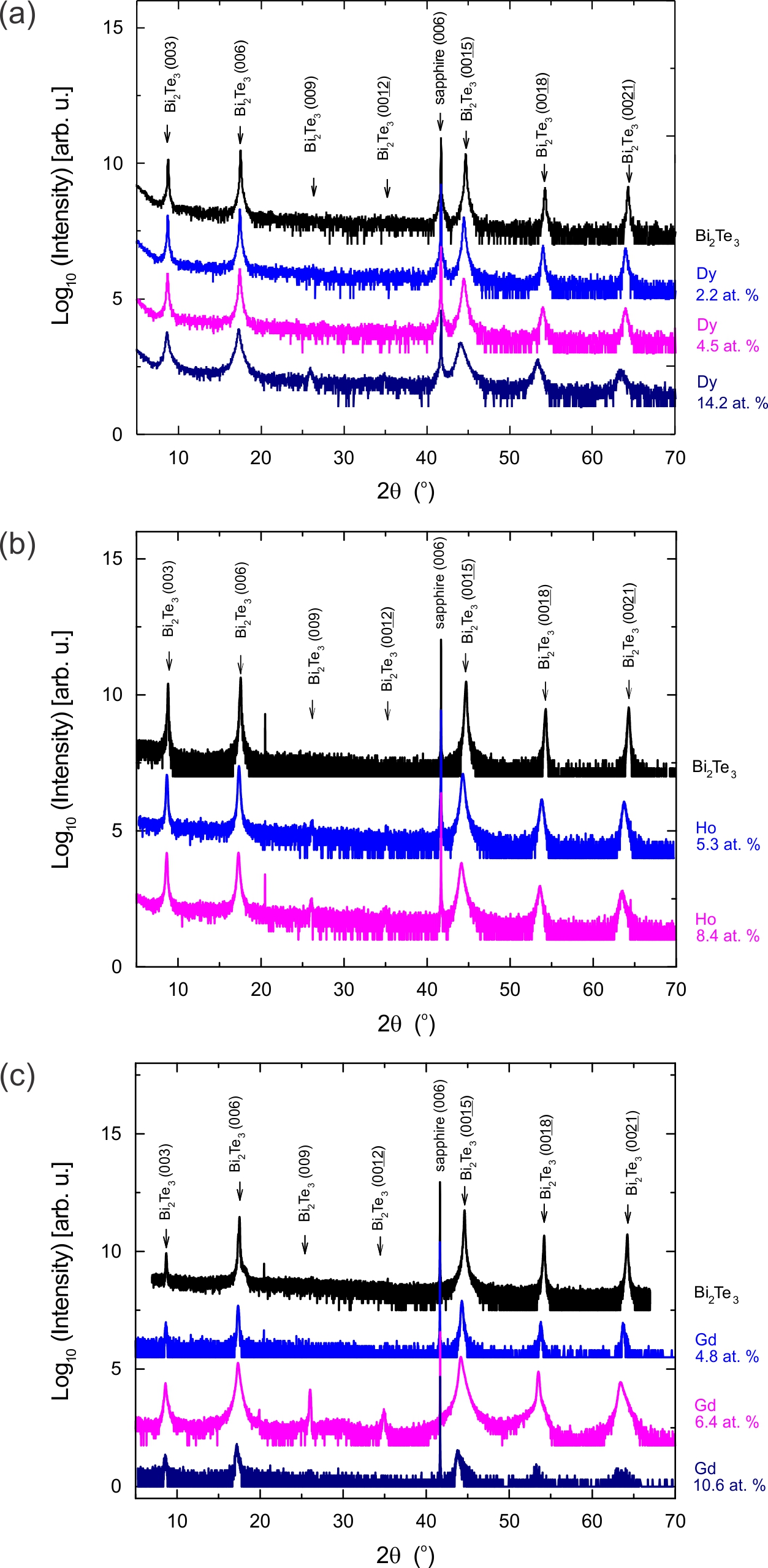


**Figure S2**. X-ray diffraction spectra for the (a) Dy-, (b) Ho-, and (c) Gd-doped Bi_2_(Se,Te)_3_ thin film samples on Al_2_O_3_ (0001). The undoped Bi_2_Te_3_ spectrum is shown at the top as a reference. The datasets have been vertically shifted for clarity.
